# Molecular Prognostic Prediction for Locally Advanced Nasopharyngeal Carcinoma by Support Vector Machine Integrated Approach

**DOI:** 10.1371/journal.pone.0031989

**Published:** 2012-03-09

**Authors:** Xiang-Bo Wan, Yan Zhao, Xin-Juan Fan, Hong-Min Cai, Yan Zhang, Ming-Yuan Chen, Jie Xu, Xiang-Yuan Wu, Hong-Bo Li, Yi-Xin Zeng, Ming-Huang Hong, Quentin Liu

**Affiliations:** 1 Department of Medical Oncology, the Third Affiliated Hospital, Sun Yat-sen University, Guangzhou, Guangdong, People's Republic of China; 2 State Key Laboratory of Oncology in South China, Cancer Center, Sun Yat-sen University, Guangzhou, Guangdong, People's Republic of China; 3 Department of Pathology, Gastrointestinal Institute, the Sixth Affiliated Hospital, Sun Yat-sen University, Guangzhou, Guangdong, People's Republic of China; 4 School of Information Science and Technology, Sun Yat-sen University, Guangzhou, Guangdong, People's Republic of China; 5 Department of Pathology, Kingmed Diagnostic Company, Guangzhou, Guangdong, People's Republic of China; 6 Clinical Trials Center and Institute of Drug Clinical Trials, Cancer Center, Sun Yat-sen University, Guangzhou, Guangdong, People's Republic of China; The Chinese University of Hong Kong, Hong Kong

## Abstract

**Background:**

Accurate prognostication of locally advanced nasopharyngeal carcinoma (NPC) will benefit patients for tailored therapy. Here, we addressed this issue by developing a mathematical algorithm based on support vector machine (SVM) through integrating the expression levels of multi-biomarkers.

**Methodology/Principal Findings:**

Ninety-seven locally advanced NPC patients in a randomized controlled trial (RCT), consisting of 48 cases serving as training set and 49 cases as testing set of SVM models, with 5-year follow-up were studied. We designed SVM models by selecting the variables from 38 tissue molecular biomarkers, which represent 6 tumorigenesis signaling pathways, and 3 EBV-related serological biomarkers. We designed 3 SVM models to refine prognosis of NPC with 5-year follow-up. The SVM1 displayed highly predictive sensitivity (sensitivity, specificity were 88.0% and 81.9%, respectively) by integrating the expression of 7 molecular biomarkers. The SVM2 model showed highly predictive specificity (sensitivity, specificity were 84.0% and 94.5%, respectively) by grouping the expression level of 12 molecular biomarkers and 3 EBV-related serological biomarkers. The SVM3 model, constructed by combination SVM1 with SVM2, displayed a high predictive capacity (sensitivity, specificity were 88.0% and 90.3%, respectively). We found that 3 SVM models had strong power in classification of prognosis. Moreover, Cox multivariate regression analysis confirmed these 3 SVM models were all the significant independent prognostic model for overall survival in testing set and overall patients.

**Conclusions/Significance:**

Our SVM prognostic models designed in the RCT displayed strong power in refining patient prognosis for locally advanced NPC, potentially directing future target therapy against the related signaling pathways.

## Introduction

Nasopharyngeal carcinoma (NPC), an Epstein-Barr virus (EBV) associated malignancy, has a remarkable racial and geographical distribution in Southeast Asia [1], [2]. Compared with the early stage patients, cancer mortality associated with disease relapse still sustained a high level in advanced NPC [3]. An accurate identification of patient prognosis will benefit this subset for developing distinct therapeutic and follow-up strategies in future.

Biomarker has been proven to be critical in predicting disease prognosis by complimenting TNM classification for risk definition [4]. More importantly, biomarkers, with dual functions for both disease monitoring and novel molecular targeting, had shed the light on personalized therapy. For example, overexpression of EGFR, which occurred in 90% of head and neck squamous cell carcinoma (HNSCC) [5], predicted an inferior patient outcome [6]. EGFR monoclonal antibody Cetuximab had demonstrated a survival benefit in combination with chemotherapy or radiotherapy for HNSCC [2], [7]. In recent BATTLE (Biomarker-Integrated Approaches of Targeted Therapy for Lung Cancer Elimination) study [8], the first large clinical trial to use tumor biomarkers to guide therapy, 11 biomarkers associated with four NSCLC molecular pathways were analyzed for directing treatment choice. The results showed that each of the four treatments (erlotinib, vandetanib, erlotinib plus bexarotene, and sorafenib) targeted potently a specific molecular signature. Thus, identifying the pathogenesis pathway related biomarkers, that not only refining the patient prognosis but also providing guidance for pathway specific target therapy, will be of great benefit for advanced cancer patients.

Data mining, including decision tree, neural networks (artificial and fuzzy), and SVM, has been applied to predict cancer patient prognosis [9], [10], [11]. Taken breast cancer and NSCLC for example, SVM had been confirmed to be a strong tool to refine the patient prognosis by integrating multi-gene profile [10], [11]. In head and neck cancer, the specific molecular pathway related biomarkers signature had not yet been characterized using the learning algorithms method based prognosis prediction model.

In the present study, we studied the expression levels of 38 markers, which represented 6 pathological signaling pathways, and 3 EBV-related serological biomarkers associated with tumorigenesis of NPC. We addressed the prognostic effect of multi-biomarkers integrated SVM models with special focus on whether SVM model could subgroup patient prognosis in head and neck cancer.

## Results

### Immunohistochemical (IHC) Staining, Univariate and ROC Curve Analysis

**Figure 1:**
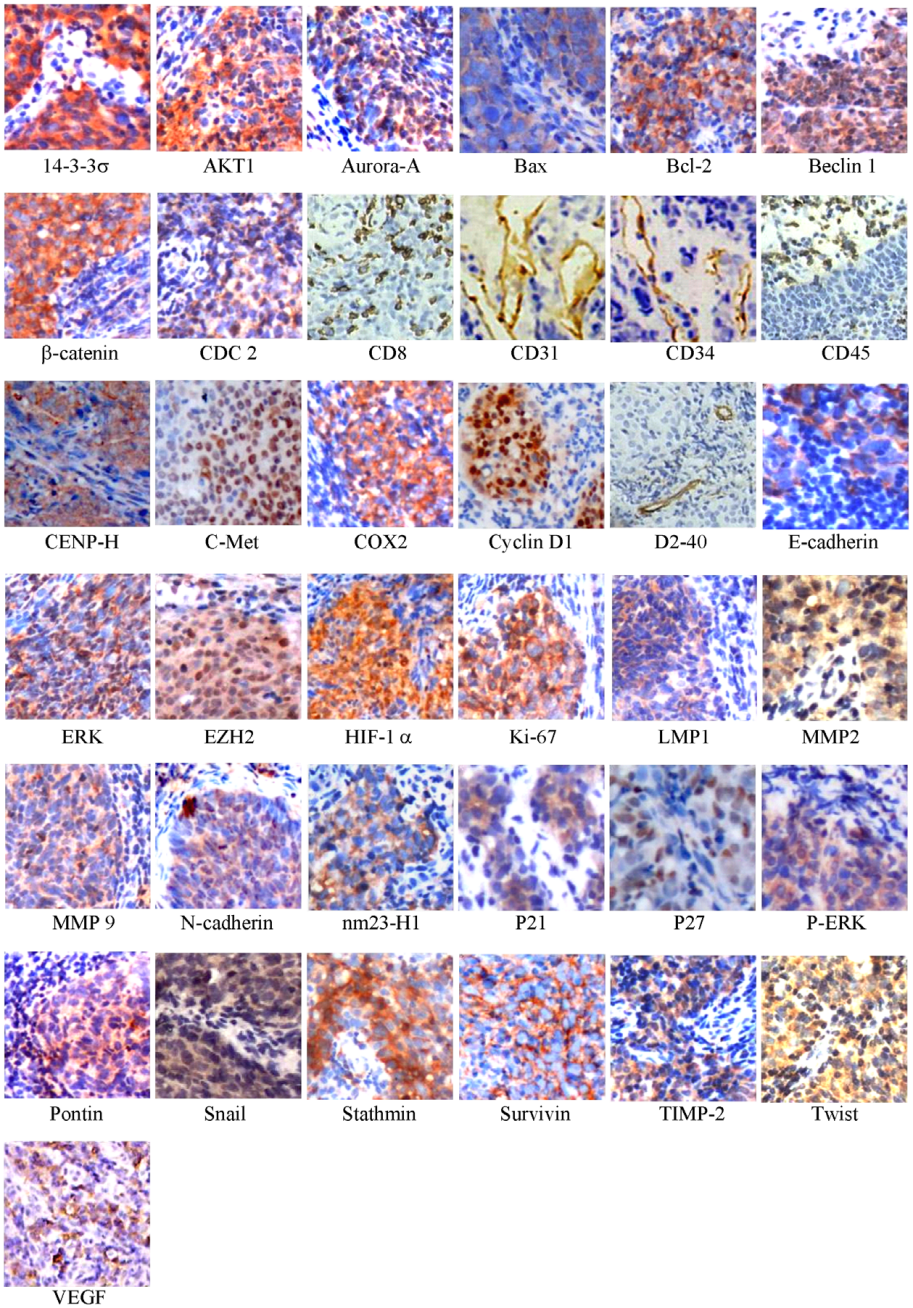
Immunohistochemical staining of tissue biomarkers in locally advanced NPC. The panel displayed the representative expression of 37 molecular biomarkers in tumor zone for locally advanced NPC (original magnification, ×400).

**Figure 2:**
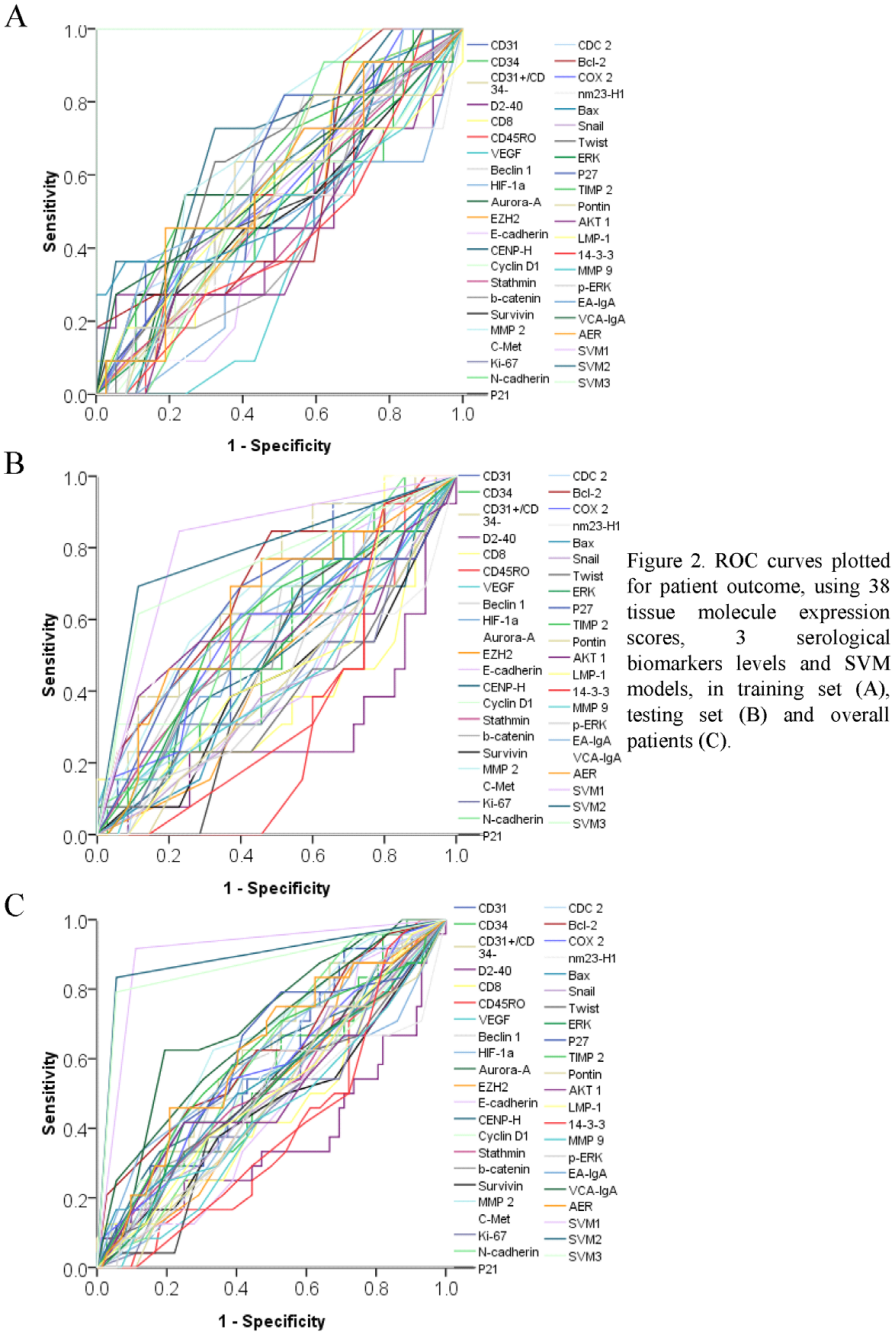
ROC curves plotted for patient outcome, using 38 tissue molecule expression scores, 3 serological biomarkers levels and SVM models, in training set (A), testing set (B) and overall patients (C). In training set (A), at each immunohistochemical staining score of 38 tissue molecules and 3 serological biomarkers, the sensitivity and specificity for the outcome being studied were plotted, thus generating a ROC curve. The score, that closest to the point with both maximum sensitivity and specificity (0.0, 1.0), was selected as the cutoff point for further analysis.

The baseline of patient clinicopathologic features of these two cohorts were displayed in Table 1. The median follow-up period was 63.8 months (range: 9.5 to 89.9 months) for overall patients. As our previous report, the IC/RT and IC/CRT subgroups displayed the similar OS (*P* = 0.783). The median overall survival was 73.9 and 70.1 months, respectively, in IC/RT and IC/CRT subgroups. The 2-year and 5-year OS was respectively 84.1% and 73.8% in IC/RT subgroup, compared with 81.8% and 72.3% in IC/CRT subset. The typical IHC staining of 38 biomarkers in these NPC samples was shown in Figure 1. As revealed in Table 2, each feature, that dichotomized by ROC curve generated cutoff point (Figure 2A), was subjected to univariate analysis. In training subgroup (48 patients), high tumor CENP-H (HR, 4.698; *P* = 0.023) and MMP 2 (HR, 3.489; *P* = 0.039) expression were associated with poor OS. In testing set, high Aurora-A (HR, 3.647; *P* = 0.021), Bcl-2 (marginal; HR, 4.423; *P* = 0.052) and VCA-IgA (HR, 3.787; *P* = 0.017) levels predicted an inferior OS. For all patients enrolled, high Aurora-A (HR, 2.872; *P* = 0.010), MMP 2 (HR, 2.942; *P* = 0.010) and VCA-IgA (HR, 2.688; *P* = 0.014) levels were correlated with worse OS. ROC curve analysis showed that SVM models had the largest area under the curve (AUC) compared with each individual AUC of 38 tissue molecules and 3 serological biomarkers (Figure 2), suggesting that SVM models was the most powerful prognostic value in refining patient outcome.

**Table 1:** Patient characteristics.

Variables	Training set (n = 48)	Testing set (n = 49)
Gender		
Male	37	41
Female	11	8
Age (Year)		
Mean ≥44.5 VS <44.5 (Range)	23 VS 25 (24 to 64)	30 VS 19 (21 to 63)
T classification		
T2	7	8
T3	22	24
T4	19	17
N classification		
N0	4	3
N1	18	18
N2	21	19
N3	5	9
TNM stage		
III	24	27
IV	24	22
Therapeutic regimen		
IC/RT	25	26
IC/CRT	23	23

Abbreviation: IC/RT, Induction chemotherapy+radiotherapy; IC/CRT, Induction chemotherapy+concurrent chemoradiotherapy.

**Table 2:** Univariate analysis of 38 tissue and 3 serological biomarkers in NPC.

Variables (>cutoff point VS ≤cutoff point)	Training set			Testing set			Overall patients		
	*P* value	HR	95% CI	*P* value	HR	95% CI	*P* value	HR	95% CI
Aurora-A, >8.5 VS ≤8.5	0.208	2.146	0.654 to 7.042	0.021	3.647	1.220 to 10.903	0.010	2.872	1.289 to 6.400
Beclin 1, >5.0 VS ≤5.0	0.097	3.074	0.815 to 11.590	0.516	1.415	0.496 to 4.037	0.110	1.919	0.862 to 4.273
HIF-1α, >7.0 VS ≤7.0	0.771	1.193	0.364 to 3.911	0.219	1.987	0.665 to 5.940	0.239	1.607	0.729 to 3.543
Bcl-2, >5.0 VS ≤5.0	0.734	0.808	0.236 to 2.760	0.052	4.423	0.989 to 19.770	0.139	1.853	0.819 to 4.195
Bax, >3.5 VS ≤3.5	0.407	1.682	0.492 to 5.754	0.490	1.471	0.492 to 4.397	0.214	1.645	0.750 to 3.610
Snail, >3.5 VS ≤3.5	0.374	1.745	0.511 to 5.963	0.273	0.556	0.195 to 1.589	0.910	0.956	0.434 to 2.106
CENP-H, >5.0 VS ≤5.0	0.023	4.698	1.243 to 17.761	0.905	1.066	0.374 to 3.042	0.084	2.025	0.909 to 4.510
COX-2, >7.0 VS ≤7.0	0.562	1.421	0.433 to 4.664	0.101	2.499	0.837 to 7.463	0.097	1.954	0.886 to 4.306
Cyclin D1, >3.5 VS ≤3.5	0.726	1.236	0.377 to 4.052	0.249	1.864	0.646 to 5.378	0.254	1.579	0.720 to 3.463
Ki-67, >5.0 VS ≤5.0	0.622	1.348	0.411 to 4.417	0.671	0.795	0.276 to 2.292	0.995	1.003	0.457 to 2.197
C-Met, >3.5 VS ≤3.5	0.245	2.074	0.607 to 7.087	0.514	1.439	0.482 to 4.296	0.188	1.731	0.765 to 3.919
MMP 2, >8.5 VS ≤8.5	0.039	3.489	1.063 to 11.452	0.140	2.394	0.750 to 7.636	0.010	2.942	1.299 to 6.663
nm23-H1, >5.0 VS ≤5.0	0.653	1.313	0.401 to 4.307	0.665	1.260	0.442 to 3.595	0.560	1.263	0.576 to 2.768
P21WAF1, >3.5 VS ≤3.5	0.530	1.464	0.446 to 4.797	0.841	1.113	0.390 to 3.174	0.520	1.294	0.590 to 2.836
Stathmin, >7.0 VS ≤7.0	0.568	0.699	0.205 to 2.388	0.368	1.653	0.554 to 4.936	0.714	1.158	0.528 to 2.538
Survivin, >2.5 VS ≤2.5	0.599	1.375	0.419 to 4.509	0.884	0.925	0.324 to 2.638	0.757	1.132	0.516 to 2.481
TIMP 2, >8.5 VS ≤8.5	0.446	1.587	0.484 to 5.202	0.133	2.254	0.781 to 6.504	0.091	1.969	0.898 to 4.318
Twist, >2.5 VS ≤2.5	0.209	2.344	0.621 to 8.840	0.572	0.739	0.259 to 2.110	0.658	1.198	0.538 to 2.668
E-cadherin, >3.5 VS ≤3.5	0.616	1.355	0.413 to 4.442	0.525	0.709	0.246 to 2.045	0.894	0.948	0.433 to 2.079
N-cadherin, >3.5 VS ≤3.5	0.379	1.737	0.508 to 5.941	0.283	1.820	0.610 to 5.431	0.172	1.768	0.781 to 4.002
β-catenin, >5.0 VS ≤5.0	0.357	0.536	0.142 to 2.022	0.514	1.423	0.493 to 4.104	0.961	0.981	0.445 to 2.161
P27, >7.0 VS ≤7.0	0.277	1.977	0.578 to 6.754	0.159	2.196	0.735 to 6.557	0.073	2.109	0.932 to 4.773
CDC 2, >5.0 VS ≤5.0	0.297	1.880	0.573 to 6.166	0.662	0.792	0.278 to 2.259	0.637	1.208	0.551 to 2.648
EZH2, >10.5 VS ≤10.5	0.732	1.230	0.375 to 4.035	0.861	0.910	0.316 to 2.623	0.928	1.037	0.473 to 2.272
ERK, >5.0 VS ≤5.0	0.910	1.074	0.314 to 3.669	0.830	1.121	0.393 to 3.198	0.729	1.150	0.522 to 2.533
p-ERK, >2.5 VS ≤2.5	0.437	1.685	0.452 to 6.280	0.986	1.011	0.321 to 3.186	0.509	1.338	0.564 to 3.176
AKT1, >5.0 VS ≤5.0	0.314	0.491	0.123 to 1.964	0.820	0.881	0.296 to 2.622	0.467	0.732	0.316 to 1.695
Pontin, >3.5 VS ≤3.5	0.708	1.255	0.383 to 4.114	0.594	0.750	0.260 to 2.162	0.966	0.983	0.446 to 2.166
MMP 9, >1.5 VS ≤1.5	0.707	0.777	0.209 to 2.896	0.201	0.482	0.157 to 1.475	0.248	0.606	0.259 to 1.418
14-3-3σ, >7.0 VS ≤7.0	0.431	0.610	0.179 to 2.086	0.063	0.365	0.126 to 1.054	0.075	0.484	0.217 to 1.077
LMP 1, >5.0 VS ≤5.0	0.633	1.336	0.407 to 4.380	0.423	0.648	0.225 to 1.871	0.856	0.929	0.422 to 2.048
CD31MVD, >12249.6 VS ≤12249.6	0.528	0.682	0.208 to 2.238	0.517	1.419	0.492 to 4.090	0.970	1.015	0.463 to 2.225
CD34MVD, >8803.7 VS ≤8803.7	0.623	1.347	0.411 to 4.416	0.581	1.347	0.467 to 3.883	0.431	1.374	0.624 to 3.026
CD31^+^/34^−^MVD, >3155.9 VS ≤3155.9	0.332	1.837	0.537 to 6.279	0.404	1.562	0.547 to 4.460	0.340	1.469	0.667 to 3.238
CD8, >62.5 VS ≤62.5	0.767	0.819	0.220 to 3.052	0.265	0.537	0.180 to 1.601	0.347	0.668	0.289 to 1.548
CD45RO, >82.5 VS ≤82.5	0.398	1.038	0.952 to 1.133	0.097	0.368	0.113 to 1.196	0.941	1.002	0.941 to 1.068
D 2–40, >95.1 VS ≤95.1	0.418	0.611	0.186 to 2.009	0.140	0.439	0.147 to 1.312	0.094	0.505	0.227 to 1.125
VEGF, >3.5 VS ≤3.5	0.129	0.281	0.054 to 1.450	0.554	1.432	0.437 to 4.693	0.469	0.709	0.280 to 1.797
EA-IgA, >1∶40 VS ≤1∶40	0.439	1.597	0.487 to 5.236	0.106	2.377	0.832 to 6.786	0.087	1.987	0.906 to 4.356
VCA-IgA, >1∶320 VS ≤1∶320	0.369	1.724	0.525 to 5.653	0.017	3.787	1.266 to 11.328	0.014	2.688	1.219 to 5.927
AER, >55.0% VS ≤55.0%	0.687	1.314	0.348 to 4.953	0.151	2/341	0.733 to 7.476	0.203	1.764	0.737 to 4.226
SVM1, 1VS 0	0.000	–	–	0.001	8.593	2.388 to 30.923	0.000	27.969	8.313 to 94.098
SVM2, 1VS 0	0.000	–	–	0.000	8.391	2.608 to 26.993	0.000	25.704	8.693 to 76.000
SVM3, 1VS 0	0.000	–	–	0.001	6.566	2.187 to 19.719	0.000	20.187	7.473 to 54.531

Abbreviation: NPC, nasopharyngeal carcinoma; MMP, matrix metalloproteinase; TIMP, tissue inhibitors of metalloproteinases; LMP, latent membrane protein; MVD, intratumoral microvessel density; VEGF, vascular endothelial growth factor; AER, anti-enzyme rate of EBV DNase-specific neutralizing antibody; SVM, support vector machines.

### SVM1 and OS

**Figure 3:**
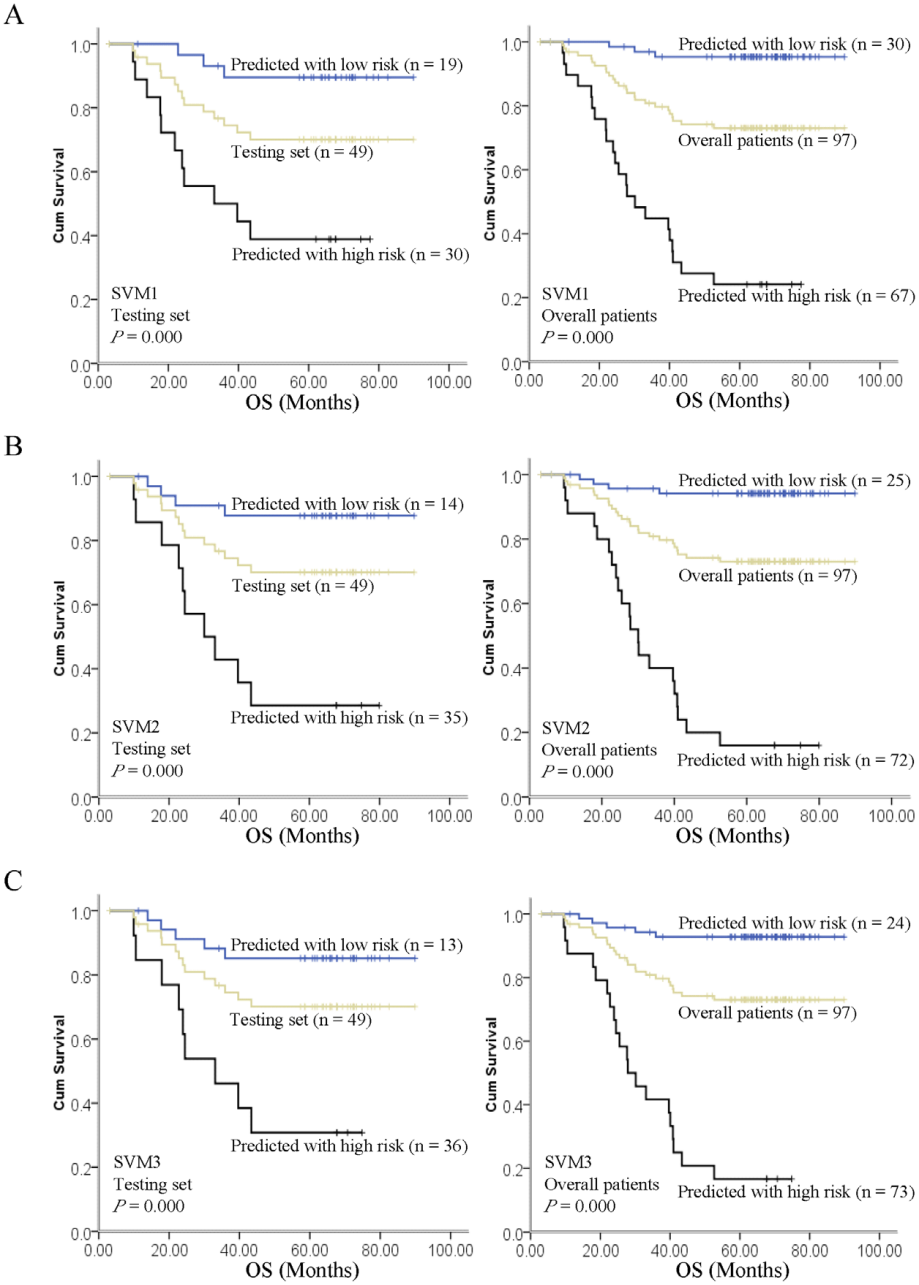
Kaplan-Meier estimated of overall survival (OS) for SVM1, SVM2 and SVM3 models identified high and low risk to death subgroups in both testing set and overall patients. For SVM1 model (A), a significant survival disadvange was observed for the high risk to death subgroup, which was identified by SVM1 model, in testing set (left panel) and overall patients (right panel). For SVM2 (B) and SVM3 model (C), a statistically OS difference was shown between high and low risk to death subgroups, which was indentified respectively by SVM2 and SVM3 model, in testing set (left panel) and overall patients (right panel).

The SVM1 model showed highly sensitivity by integrating the expression levels of 7 tissue molecular biomarkers, including of Aurora-A, Beclin 1, Ki-67, N-cadherin, nm23-H1, P27 and TIMP 2. After educating the model in the training set, we identified 19 patients with high risk to death and 30 patients with low risk at testing set. The 5-year OS of the subgroup with high risk to death was 38.9% compared with 89.5% in low risk subset (Figure 3A, *P*<0.0001). Specifically, the predictive value of SVM1 in sensitivity, specificity, positive predictive value, negative predictive value, and overall accuracy were 78.6%, 77.1%, 57.9%, 90.0% and 77.6%, respectively. Cox multivariate regression analysis confirmed that SVM1 model was indeed the significant independent predictive model for patient risk to death (Table 3; HR, 42.275; 95% CI, 2.474 to 722.425; *P* = 0.010).

**Table 3:** Multivariate Cox Regression Analysis of the SVM1, SVM2 and SVM3 Models in Testing Set and Overall Patients.

Variables (>cutoff point VS ≤cutoff point)	SVM1				SVM2				SVM3			
	Testing set		Overall patients		Testing set		Overall patients		Testing set		Overall patients	
	Hazard Ratio (95% CI)	*P* value	Hazard Ratio (95% CI)	*P* value	Hazard Ratio (95% CI)	*P* value	Hazard Ratio (95% CI)	*P* value	Hazard Ratio (95% CI)	*P* value	Hazard Ratio (95% CI)	*P* value
Aurora-A, >8.5 VS ≤8.5	1.084 (0.150 to 7.811)	0.936	0.278 (0.085 to 0.911)	0.035	1.795 (0.252 to 12.802)	0.559	0.806 (0.217 to 2.992)	0.748	0.116 (0.004 to 3.768)	0.225	0.407 (0.096 to 1.734)	0.224
Beclin 1, >5.0 VS ≤5.0	1.240 (0.279 to 5.510)	0.778	1.255 (0.475 to 3.314)	0.647	9.101 (0.686 to 120.797)	0.094	1.581 (0.478 to 5.232)	0.453	4.374 (0.386 to 49.593)	0.234	1.239 (0.411 to 3.730)	0.703
Ki-67, >5.0 VS ≤5.0	0.239 (0.037 to 1.565)	0.135	0.549 (0.217 to 1.391)	0.206	0.534 (0.035 to 8.207)	0.653	1.674 (0.391 to 7.165)	0.488	0.476 (0.043 to 5.289)	0.546	1.588 (0.400 to 6.303)	0.511
N-cadherin, >3.5 VS ≤3.5	0.866 (0.187 to 4.017)	0.854	0.656 (0.227 to 1.893)	0.435	3.267 (0.216 to 49.508)	0.393	2.705 (0.640 to 11.424)	0.176	5.211 (0.457 to 59.465)	0.184	2.856 (0.713 to 11.445)	0.138
nm23-H1, >5.0 VS ≤5.0	2.050 (0.345 to 12.199)	0.430	1.291 (0.448 to 3.718)	0.636	3.633 (0.469 to 28.163)	0.217	0.702 (0.199 to 2.474)	0.582	0.743 (0.080 to 6.896)	0.794	0.829 (0.253 to 2.722)	0.757
TIMP 2, >8.5 VS ≤8.5	0.997 (0.226 to 4.405)	0.997	1.141 (0.464 to 2.807)	0.774	0.271 (0.034 to 2.144)	0.216	0.607 (0.178 to 2.071)	0.426	0.086 (0.004 to 2.075)	0.131	1.097 (0.338 to 3.558)	0.878
P27, >7.0 VS ≤7.0	1.706 (0.485 to 6.007)	0.405	2.235 (0.815 to 6.127)	0.118					49.267 (0.428 to 5672.637)	0.108	1.919 (0.561 to 6.570)	0.299
EZH2, >10.5 VS ≤10.5					7.050 (0.702 to 70.789)	0.097	1.270 (0.437 to 3.695)	0.660	2.490 (0.384 to 16.143)	0.339	1.369 (0.432 to 4.339)	0.594
Cyclin D1, >3.5 VS ≤3.5					0.076 (0.003 to 2.109)	0.129	0.325 (0.072 to 1.466)	0.144	0.168 (0.011 to 2.503)	0.195	0.381 (0.084 to 1.730)	0.211
MMP 2, >8.5 VS ≤8.5					0.601 (0.011 to 32.454)	0.803	0.962 (0.262 to 3.538)	0.954	6.801 (0.159 to 290.224)	0.317	1.422 (0.387 to 5.224)	0.596
Bcl-2, >5.0 VS ≤5.0					0.707 (0.032 to 15.786)	0.827	1.779 (0.476 to 6.641)	0.392	0.645 (0.027 to 15.350)	0.786	1.441 (0.395 to 5.259)	0.580
14-3-3σ, >7.0 VS ≤7.0					0.239 (0.022 to 2.589)	0.239	0.356 (0.102 to 1.240)	0.105	0.212 (0.014 to 3.331)	0.270	0.648 (0.190 to 2.202)	0.487
EA-IgA, >1∶40 VS ≤1∶40					0.085 (0.003 to 2.268)	0.141	0.149 (0.018 to 1.243)	0.079	0.402 (0.021 to 7.634)	0.544	0.167 (0.023 to 1.228)	0.079
VCA-IgA, >1∶320 VS ≤1∶320					79.076 (1.136 to 5505.569)	0.044	34.620 (2.903 to 412.842)	0.005	140.873 (1.602 to 1.238×10^4^)	0.030	38.911 (3.564 to 424.812)	0.003
AER, >55.0% VS ≤55.0%					0.441 (0.048 to 4.065)	0.470	0.520 (0.121 to 2.234)	0.379	0.676 (0.070 to 6.515)	0.735	0.828 (0.220 to 3.112)	0.780
Pontin, >3.5 VS ≤3.5					0.178 (0.032 to 1.005)	0.051	0.329 (0.093 to 1.160)	0.084	0.526 (0.065 to 4.290)	0.549	0.357 (0.114 to 1.111)	0.075
SVM1, 1VS 0	11.015 (1.773 to 68.414)	0.010	70.745 (16.161 to 309.695)	0.000								
SVM2, 1VS 0					653.763 (3.550 to 1.204×10^5^)	0.015	145.080 (17.776 to 1184.065)	0.000				
SVM3, 1VS 0									266.381 (0.866 to 8.196×10^4^)	0.056	113.594 (16.513 to 781.461)	0.000

By summarizing the training and testing set as a group, we identified 30 patients with high risk and 67 patients with low risk to death. The 5-year OS of the patients with high risk to death was 24.1% compared with 95.3% in low risk subgroup (Figure 3A, *P*<0.0001). Specifically, the predictive value of SVM1 in sensitivity, specificity, positive predictive value, negative predictive value, and overall accuracy were 88.0%, 81.9%, 62.9%, 95.2% and 83.5%, respectively. Cox multivariate regression analysis confirmed that SVM1 model was the significant independent predictive model for patient risk to death (Table 3; HR, 320.826; 95% CI, 36.705 to 2804.256; *P*<0.0001). Moreover, a prognostic effect on age, Aurora-A, Ki67 and P27 were also observed in overall patients, though with relatively low HR (Table 3). The clinical features, including of gender, TNM stage as well as therapeutic regimens, and other molecular biomarkers however failed to prove any prognostic value.

### SVM2 and OS

The SVM2 model showed high specificity by grouping the expression levels of 12 tissue molecular biomarkers (nm23-H1, Pontin, cyclin D1, N-Cadherin, 14-3-3σ, Ki-67, Aurora-A, Bcl-2, Beclin 1, MMP 2, EZH2 and TIMP 2) and 3 EBV-related serological biomarkers (EA-IgA, VCA-IgA and AER). After educating the model in the training set, we identified 14 patients with high risk to death and 35 patients with low risk at testing set individually. The 5-year OS of the subset with high risk to death was 28.6% compared with 87.8% in low risk subgroup (Figure 3B, *P*<0.0001). In detail, the predictive value of SVM2 in sensitivity, specificity, positive predictive value, negative predictive value, and overall accuracy were 71.4%, 88.6%, 71.4%, 88.6% and 83.7%, respectively. Cox multivariate regression analysis confirmed that SVM2 model was indeed the significant independent predictive model for patient risk to death (Table 3; HR, 6055.528; 95% CI, 2.718 to 1.349×10^7^; *P* = 0.027).

By summarizing the training and testing set as a group, we identified 25 patients with high risk and 72 patients with low risk to death. The 5-year OS of the patients with high risk to death was 16.0% compared with 94.2% in low risk subgroup (Figure 3B, *P*<0.0001). Specifically, the predictive value of SVM2 in sensitivity, specificity, positive predictive value, negative predictive value, and overall accuracy were 84.0%, 94.5%, 84.0%, 94.4% and 91.8%, respectively. Cox multivariate regression analysis confirmed that SVM2 model was the significant independent predictive model for patient risk to death (Table 3; HR, 346.294; 95% CI, 24.742 to 4846.721; *P*<0.0001). In addition, Cyclin D1, EA-IgA and VCA-IgA were also the independent prognostic factors in overall patients, though with relatively low HR (Table 3).

### SVM3 and OS

The SVM3 model, that incorporating SVM1 with SVM2, was subjected to refine patient risk to death when risk definition discrepancy was confronted at SVM1 and SVM2. In SVM3 model, we integrated the expression level of 13 tissue molecular biomarkers (nm23-H1, Pontin, cyclin D1, N-Cadherin, 14-3-3σ, Ki-67, Aurora-A, Bcl-2, Beclin 1, MMP 2, EZH2, TIMP 2 and P27) and 3 EBV-related serological biomarkers (EA-IgA, VCA-IgA and AER). As shown in Figure 3C, we identified 13 patients with high risk and 36 patients with low risk to death at testing set. The 5-year OS of the subset with high risk to death was 30.8% compared with 85.2% in low risk subgroup (Figure 3C, *P*<0.0001). Specifically, the predictive value of SVM3 in sensitivity, specificity, positive predictive value, negative predictive value, and overall accuracy were 64.3%, 88.6%, 69.2%, 86.1% and 81.6%, respectively. Cox multivariate regression analysis confirmed that SVM3 model was indeed the independent predictive model for patient risk to death (Table 3; HR, 1401.433; 95% CI, 0.883 to 2.223×10^6^; *P* = 0.054).

By summarizing the training and testing set as a group, we identified 24 patients with high risk and 73 patients with low risk to death. The 5-year OS of the patients with high risk to death was 16.7% compared with 92.8% in low risk subgroup (Figure 3C, *P*<0.0001). Specifically, the predictive value of SVM3 in sensitivity, specificity, positive predictive value, negative predictive value, and overall accuracy were 88.0%, 90.3%, 75.9%, 95.6% and 89.7%, respectively. Cox multivariate regression analysis confirmed that SVM3 was an independent predictive model for patient risk to death (Table 3; HR, 540.456; 95% CI, 33.336 to 8761.995; *P*<0.0001). Additionally, age, P27 and VCA-IgA showed prognostic effect for overall patients, though with the lower HR (Table 3).

## Discussion

The important challenge complementing the anatomic TNM staging prognostication is to integrate the nonanatomic molecular biomarkers [12]. Indeed, circulating serological and tissue molecular prognostic factors were currently used for predicting cancer patient outcome individually. Here, we examined the expression levels of 38 tissue molecular biomarkers representing 6 pathological signaling pathways and 3 EBV-related serological biomarkers for further characterizing their prognostic value by constructing the SVM models in a randomized controlled trial. By integrating 16 biomarkers that displayed higher predictive values, we designed 3 SVM prognosis models. Our finding demonstrated that those 3 SVM models showed the powerful efficacy in defining patient risk to death individually, indicating the promising clinical usage in future therapeutic and follow-up management.

Accurate characterization of patient outcome, that not only permits treatment to be individualized but also improves patient follow-up economic benefit cost ratio, is markedly important for locally advanced NPC. Biomarkers that aberrantly expressed in tissue or circulation have been proven to be critical in guiding treatment selection and predicting disease prognosis [4], [12]. Both the single biomarker reflecting the cancer phenotype in a microscopical manner and the TNM stage system predicting patient outcome in a macroscopical manner showed however a limited predictive power for individual outcome. In the present study, we designed a SVM model by integrating the expression levels of several tissues molecular biomarkers and NPC specific serological biomarkers to refine patient risk to death individually. We thus raised three key clinical implications of this SVM based prognostic model for locally advanced NPC: i) the molecular biomarkers included in this study were detected by IHC and ELISA and thus might be readily adaptable to clinical practice; ii) patients with inconsistent definition of risk to death between SVM1 and SVM2 models would be subjected to SVM3 for further determination. iii) the therapeutic regimen for advanced NPC might be redirected for the particular subgroup according to the SVM risk definition. Specifically, the patients with low risk definition could receive routine therapeutic regimen to avoid the serious side effect of intensive treatment modality. However, for patients with high risk definition, the standard chemoradiotherapy might not be sufficient. The target agent that specific to the particular molecular biomarker [13], and more aggressive chemotherapy regimen may be employed to maximize the therapeutic benefit.

In comparison with other data mining methods [14], such as neural networks (artificial and fuzzy) [15], clustering [16], genetic algorithms [17] and decision trees [18], SVM performs classification by constructing an *N*-dimensional hyperplane that optimally separates the data into two categories [19]. This feature thus presented great priority in predicting cancer patients prognosis that with two classifications (death VS alive). Additionally, Newman-Keuls test was used to deal with multiple comparisons that raised by multiple variables included in this study, ensuring the rational IHC score for further SVM analysis. More importantly, the higher generalization ability made SVM could train the model with limited cases by grouping several efficient features. Here, we designed the SVM models for advanced NPC by integrating TNM stage, tissue molecular features (nm23-H1, Pontin, cyclin D1, N-Cadherin, 14-3-3σ, Ki-67, Aurora-A, Bcl-2, Beclin 1, MMP 2, EZH2, TIMP 2, COX 2 and P27) along with EBV-related biomarkers (AER, EA-IgA and VCA-IgA), which reflected each patient tumorigenesis phenotype not only in macroscopic but also in microcosmic aspect. Thus, these multibiomarkers based models would provide more powerful efficacy in prediction of patient outcome. Indeed, our finding confirmed that the SVM models had strong ability in refining patient risk to death individually (Figure 3, high risk VS low risk: SVM1 24.1% VS 95.3%, SVM2 16.0% VS 94.2%, SVM3 16.7% VS 92.8%). However, we also observed the inconsistence in predicting patient outcome among SVMs in testing set regarding to age, Aurora-A, P27 and VCA-IgA. Taken Aurora-A for example, *P* value were 0.608, 0.683 and 0.098 for SVM1, SVM2 and SVM3, respectively. The underlying reasons might lie in the small cohort size in testing set since the significant prognostic value was observed when the cohort combined both training set and testing subgroup.

Taken together, our study demonstrated that multibiomarkers integrated SVM models led to more precise risk definition, offering a promising and individualized selection for future therapeutic regimen.

## Methods

### Patients

The 408 locally advanced NPC patients (Stage III and IV_a_) were enrolled in a randomized controlled trial (RCT) designed for therapeutic as well as SVM-biomarker study from August 2002 to April 2005 [20]. In the therapeutic study, the therapeutic effect of induction chemotherapy+radiotherapy (IC/RT) was compared to induction chemotherapy+concurrent chemoradiotherapy (IC/CRT). In this biomarker study, randomized 103 patients (50 IC/CRT+53 IC/RT) were selected for multi-biomarkers-SVM prognosis analysis. Excluding 6 patients (4 IC/CRT+2 IC/RT) lost to 5-year follow-up, 97 patients (46 IC/CRT+51 IC/RT) were enrolled in this study. The baseline of patient clinicopathologic features of these two cohorts were displayed in Table 1. Of these 97 patients, randomly selected 48 patients (25 IC/RT+23 IC/CRT) were used as training set for SVM model education and the rest of 49 patients (26 IC/RT+23 IC/CRT) served as testing set. The cancer stage was defined according to the 1992 NPC staging system of China [21], [22]. This study was approved by the Clinical Ethics Review Board at Cancer Center of Sun Yat-sen University, and written informed consent was obtained from all patients at their recruitment.

### Patient eligibility

In this RCT, strict eligibility criteria protocol was employed as following: pathological confirmed as nonkeratinizing or undifferentiated carcinoma of nasopharynx (World Health Organization types of II or III); aged 18–65 years; performance status score: 0–2; clinical stage: III-IV_a_; leukocyte count (WBC) ≥4.0×10^9^/L and platelet ≥100.0×10^9^/L; total bilirubin (TBIL) and alanine aminotransferase (ALT) <2× the upper limit of normal value; creatinine (Cr) <1.5× the upper limit of normal value. Patients were excluded from this RCT with the following exclusion criteria: uncontrolled infection; previously received any anticancer therapy; pregnancy and lactation; prior malignancy; unsuitable for chemotherapy due to deficiency of liver, kidney, lung and heart.

The routine staging workup comprised of a detailed clinical examination of the head and neck, fiberoptic nasopharyngoscopy, magnetic resonance imaging (MRI) of the entire neck from the base of the skull, chest radiography, abdominal sonography, a complete blood count, and a biochemical profile. New Drug Statistical Treatment 8.0 software was employed to generate a random number table for further patient assignment.

### Oncologic treatment

In IC/RT subset, patients received two cycle of floxuridine+carboplatin (floxuridine 750 mg/m^2^, d1–5; carboplatin AUC = 6) chemotherapy and underwent radiotherapy thereafter at one week interval. In IC/CRT subgroup, one week after completion of two cycle floxuridine+carboplatin (floxuridine 750 mg/m^2^, d1–5; carboplatin AUC = 6), patients received radiotherapy and concurrent carboplatin (AUC = 6) chemotherapy on day 7, 28 and 49, respectively.

Prior to and after the carboplatin infusion, 1000–1500 ml normal saline, 20 g (250 ml) and normal saline 2000–3000 ml were respectively given to patients. Aheading of drug infusion, the 5-hydroxytryptamine-3 receptor antagonists and dexamethasone (20 mg) were used to guard against vomiting. For patients with serious myelosuppression, the chemotherapy schedule would be delayed to the serological leukocyte counts ≥3.0×10^9^/L and platelet count ≥100.0×10^9^/L. The carboplatin dose adjustment was based on the level of posttreatment creatinine clearance. When posttreatment serological creatinine clearance ≥60 mL/min, the original regimen could be maintained at the next cycle of chemotherapy. When the creatinine clearance decreased to 40–59 mL/min, a reduction of 50% carboplatin dose was required for the next cycle of chemotherapy. Once the serum creatinine clearance was less than 40 mL/min, carboplatin should be removed at the next cycle of chemotherapy.

The traditional Co^60^ γ-ray or linear accelerator 6–8 MV photon based two-dimensional technique was administered for radiotherapy. The radiation fields were determined by the extension of the tumor and local regional cervical lymph node invasiveness. The target radiation fields, including the tumor and a 2-cm marginal extension in all directions, obtained at least 90% of the mid-depth central axis dose. During the first course, two lateral opposing faciocervical portals were exposed to 36–40 Gy irradiation. At the second course, facio-cervical splitting portals course was employed. When the oropharynx was invaded, the facio-cervical portals would be used in these 2 courses, followed by 8–12 Mev electric beam irradiation at the posterocervical triangular regions. The anterior nasal region (6–8 Gy) or the parapharyngeal region (6–8 Gy) would be irradiated for subset with regional tumor invasion.

The accumulated radiation dose of 68–72 Gy, with 2 Gy daily fractions and 5 days per week, was given to the primary tumor. Additional boosted 8 to 12 Gy would be delivered to subgroup with residual tumor and destructed skull base. The neck region obtained 50 to 70 Gy radiation according to the extent of the lymph node tumorigenic invasiveness. For lymph node negative and positive invaded necks, 50 Gy and 60 to 70 Gy radiation would respectively be given.

### Immunohistochemical (IHC) staining and EBV-related serological antibodies assay

Both tissue microassays and IHC were performed as previously described [23], [24]. The candidate biomarkers consisted of reported prognostic markers with high predictive value and a number of key tumorigenesis signaling pathways related molecules [12]. A total of 38 biomarkers (Figure 1) representing 6 pathological signaling pathways related to NPC disease progression, consisting of cell cycle: Cyclin D1 (Cell Signaling, #2978, 1∶200 dilution), 14-3-3σ (Santa Cruz, SC-100638, 1∶200 dilution), Aurora-A (Upstate, 1∶200 dilution), CENP-H (Santa Cruz, SC-22792, 1∶200 dilution), Stathmin (Cell Signaling, #3352, 1∶200 dilution), P21WAF1 (Santa Cruz, SC-817, 1∶200 dilution), CDC2 (Santa Cruz, SC-53, 1∶200 dilution), P27 (Millipore, clone Y236, 1∶200 dilution), ERK (Santa Cruz, SC-94, 1∶200 dilution), p-ERK (Santa Cruz, SC-7383, 1∶100 dilution), Ki-67 (Santa Cruz, SC-23900, 1∶50 dilution); migration & invasion: E-Cadherin (Cell Signaling, #4065, 1∶50 dilution), β-catenin (Millipore, MAB2081, 1∶100 dilution), N-Cadherin (Upstate, clone 13A9, 1∶200 dilution), Snail (Abcam, ab70983, 1∶50 dilution), Twist (Santa Cruz, SC-102032, 1∶50 dilution), c-Met (Santa Cruz, SC-161, 1∶200 dilution), nm23-H1 (Santa Cruz, SC-56928, 1∶200 dilution); tumor microenvironment: HIF-1α Millpore, MAB5382, 1∶200 dilution), COX2 (Santa Cruz, SC-58344, 1∶200 dilution), MMP-2 (Santa Cruz, SC-53630, 1∶200 dilution), MMP-9 (Santa Cruz, SC-6840, 1∶200 dilution), TIMP-2 (Santa Cruz, SC-21753, 1∶200 dilution), VEGF (MaiXin, 1∶200 dilution), CD31 (MaiXin, 1∶200 dilution) microvessel density (MVD), CD34 (MaiXin, 1∶200 dilution) MVD, CD31^+^/CD34^−^ MVD, CD8 (MaiXin, 1∶200 dilution), CD45RO (MaiXin, 1∶200 dilution) and D2–40 (MaiXin, 1∶200 dilution); apoptosis & autophagy: Bax (Santa Cruz, SC-7480, 1∶200 dilution), Bcl-2 (Santa Cruz, SC-7382, 1∶50 dilution), Survivin (Santa Cruz, SC-47750, 1∶100 dilution), AKT 1 (Cell Signaling, #4685, 1∶100 dilution), Pontin (Cell Signaling, #8959, 1∶100 dilution), Beclin 1 (Santa Cruz, SC-11427, 1∶200 dilution); epigenetic related molecule EZH2 (Cell Signaling, #4905, 1∶200 dilution) and EBV related molecule LMP 1 (Santa Cruz, SC-57721, 1∶200 dilution), were tested in this study. A negative control was utilized by changing the specific primary antibody with non-immune serum immunoglobulins at the 1∶200 dilutions. Serological EBV related antibodies, EA-IgA, VCA-IgA and anti-enzyme rate (AER) of EBV DNase-specific neutralizing antibody, were tested prior to oncologic treatment by ELISA method [25], [26]. Antibody testing of each used antibody was done prior to the IHC staining.

### Semi-quantitative assessment of IHC

The expression profile of each biomarker was evaluated by combined assessment of staining intensity and extent as we previously described [13], [24]. Immunohistochemical (IHC) staining was evaluated and scored by two independent pathologists (X.-J.F. & J.X.) blindly to clinical follow-up data. The third pathologist will arbitrate the discrepancy arose between these two pathologists. Totally, the ratio of complete agreement of the overall score reached to 87%. The MVD were evaluated by counting CD31^+^ capillaries, CD34^+^ capillaries, CD31^+^/CD34^−^ capillaries and D2–40 (lymphangial specific marker) in the three most vascularized areas (“hotspots”) [27], [28]. The immune microenvironment reactivity was assessed by counting positive stained CD8 and CD45RO T cells in the three “hotspots” [29].

### Selection of cutoff score for each biomarker “positive” expression

The receiver operating characteristic (ROC) curve analysis was subjected to the selection of cutoff score in the training set as we previously reported [24]. Briefly, the sensitivity and specificity for patient outcome being studied at each score were plotted to generate a ROC curve. The score localized closest to the point at both maximum sensitivity and specificity, the point (0.0, 1.0) on the curve, was selected as the cutoff score that might be correctly classified patient outcome as death or alive.

### Clinical outcome assessment

The patients in this RCT were all followed up with strict protocol. After the completion of therapy, patients were observed at 3-month intervals during the first 3 years and at 6-month intervals thereafter. The latest date of each patient being followed up was May 15 2010, ensuring the accurate 5-year survival condition of each patient was obtained and readily for further SVM analysis. The 5-year survival condition was defined as death or alive at the appointed date of 5 years post-diagnosis. Overall survival (OS) was defined as the time from diagnosis to the date of death or censored at the latest date.

### Sample size estimation

Given the robust capacity of SVM prognostication model in optimally separating the data into two categories, a relative small sample size might be enough to achieve the goal of powerful prognosis prediction. In this study, the STATA COX regression was used to estimate the sample size based on 38 biomarkers expression level. In light of the 24.2% OS events probability in therapeutic regimen RCT, a total of 95 cases were required to achieve 90% power for a 5% significance level assuming the OS HR increased >ten-fold for SVMs model. When considering the subgroup that might loss to follow-up, the cases size was further enlarged to 103.

### Support vector machines (SVM) model construction

In this study, we considered the patient prognosis as a two-class pattern classification (death VS alive). We employed a vector 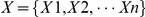 to denote the pattern of *n* components for a patient. In our binary classification, patient who survived for more than 5 years was denoted by 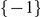 whereas 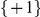 represented the patient survived less than 5 years. The overall patients were randomly divided into two subgroups: *training set* that was employed to construct decision function *D* (•), and *testing set* was used to test the predictive accuracy of decision function.

The main procedure for SVM classification involved two steps. Firstly, the input feature vector X is mapped into a higher dimensional space *H* through an underling nonlinear mapping *ϕ* 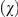. Secondly, the linear classification is applied in this mapping space. A SVM decision function 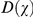 can be rewritten as 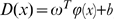, where parameter 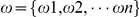 denotes the *support vector*. The unknown parameter ω could be obtained through minimization of the following *structural risk* function: 
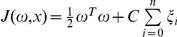
 (1), 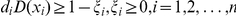. The value of 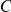
is a user-specified positive parameter, and ξ*_i_* are slack variables. Given the two classes are separable, minimizing the structural risk in (1) contributes to the maximal separating margin between these two classes.

In this study, the performance of classification was calculated using the following loss function: 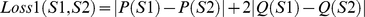, where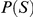 and 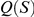 denoted the overall accuracy and sensitivity for set S, respectively. The definition of 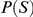 and 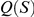 was 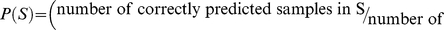 
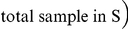
 (2), 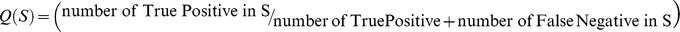.

To maximize the area under ROC curve, we also defined another loss function based on ROC parameter as following: 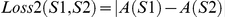 (3), where 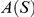 indicated the area under ROC curve for the testing set S.

The classical RBF kernel function 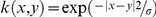 was used in SVM model construction. To find optimal parameters of SVM model, including kernel size σ and regularization parameter C, standard Leave-one-out cross-validation was employed to search over a grid 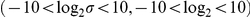.

### Statistical analysis

The multivariate Cox proportional hazards model was utilized to estimate the hazard ratio (HR) and 95% confidence interval (CI). The survival probabilities difference between patients subsets in OS were determined by Kaplan-Meier analysis and log-rank tests. A two-tailed *P*<0.05 was considered statistically significant. Statistical analysis was performed using SPSS v. 17.0 (SPSS, Inc., Chicago, IL).
